# Cost-effectiveness of a canine visceral leishmaniasis control program in Brazil based on insecticide-impregnated collars

**DOI:** 10.1590/0037-8682-0680-2020

**Published:** 2020-12-11

**Authors:** Tália Machado de Assis, André Luís Ferreira de Azeredo-da-Silva, Gláucia Cota, Marília Fonseca Rocha, Guilherme Loureiro Werneck

**Affiliations:** 1 Centro Federal de Educação Tecnológica de Minas Gerais, Contagem, MG, Brasil.; 2 Fundação Oswaldo Cruz,Instituto René Rachou, Pesquisas Clínicas e Políticas Públicas em Doenças Infecciosas e Parasitárias, Belo Horizonte, MG, Brasil.; 3 Instituto para Avaliação de Tecnologias em Saúde, Porto Alegre, RS, Brasil.; 4 Universidade Federal do Rio Grande do Sul, Porto Alegre, RS, Brasil.; 5 Universidade Estadual de Montes Claros, Centro de Ciências Biológicas e da Saúde, Departamento de Saúde Mental e Saúde Coletiva, Montes Claros, MG, Brasil.; 6 Secretaria Municipal de Saúde, Centro de Controle de Zoonoses, Programa de Controle das Leishmanioses, Montes Claros, MG, Brasil.; 7 Universidade do Estado do Rio de Janeiro, Instituto de Medicina Social, Departamento de Epidemiologia, Rio de Janeiro, RJ, Brasil.

**Keywords:** Collars, Visceral leishmaniasis, Cost-effectiveness

## Abstract

**INTRODUCTION:**

The use of insecticide-impregnated dog collars is a potentially useful tool for the control of visceral leishmaniasis. The objective of the present study was to perform a cost-effectiveness analysis of a program based on insecticide-impregnated collars compared to traditional visceral leishmaniasis control strategies used in Brazil.

**METHODS:**

A cost-effectiveness analysis was performed from the perspective of the Unified Health System, using data from the Visceral Leishmaniasis Control Program implemented in the municipality of Montes Claros, Minas Gerais. The direct costs of the three control strategies, which were 1) canine infection screening + sacrifice, 2) residual chemical control of the vector, and 3) insecticide-impregnated dog collars (Scalibor^®^), were evaluated over the two-year study period.

**RESULTS:**

The total cost of the program in the area subjected to the traditional control strategies (strategies 1 and 2; control area) was R$ 1,551,699.80, and in the area subjected to all three control strategies (intervention area), it was R$ 1,898,190.16. The collar program was considered highly cost-effective at preventing canine visceral leishmaniasis (incremental cost-effectiveness ratio of approximately R$ 578 per avoided dog sacrifice).

**CONCLUSIONS:**

These results provide support for the decision by the Brazilian Ministry of Health in 2019 to provide insecticide-impregnated collars for the control of canine visceral leishmaniasis in a pilot project.

## INTRODUCTION

Visceral leishmaniasis (VL) is a serious parasitic disease in Brazil caused by the protozoan *Leishmania infantum* and transmitted through the bite of insects of the genus *Lutzomyia*. *Lutzomyia longipalpis* is the main transmitting species and dogs are the main reservoir in urban environments^1^ **.** The disease is rapidly expanding and urbanizing in the country, and controlling it is considered a public health challenge^2^ ^-^ ^3^.

The strategy of the VL Surveillance and Control Program of the Ministry of Health (MH) in Brazil is centered on interventions aimed at patients, insect vectors, and the canine population. The following actions are recommended: diagnosis and treatment of human cases, vector control through insecticides and the control of canine infection, and sacrifice of seropositive dogs^4^. In general, the program has shown low effectiveness in interrupting VL transmission^5^ ^-^ ^6^; therefore, new tools for disease control are necessary.

The use of insecticide-impregnated dog collars is a potentially useful tool for the control of VL^7^. It acts by repelling and killing insects, which reduce vector contact with dogs^8^ for up to eight months^9^, in addition to reducing flea^7^ and tick infestation^10^.

Between 2011 and 2016, Alves et al. (2020)^11^ evaluated the effectiveness of collars impregnated with 4% deltamethrin (Scalibor^®^) used in addition to control measures already recommended in Brazil (vector control with insecticides, monitoring of canine infection, and subsequent sacrifice of seropositive dogs). In total, 22 sampling units were studied in the cities of Teresina (Piauí), Fortaleza, Canindé, Maracanaú and Eusébio (Ceará), Araguaína (Tocantins), and Montes Claros (Minas Gerais), with over 120,000 dogs collared in the intervention areas and over 90,000 dogs examined in the control areas. In that study, the use of collars was associated with a significant reduction (approximately 50%) in the prevalence of canine infection compared to control measures. This result suggests that collaring adds a benefit to the control measures already recommended in Brazil; however, before its large-scale implementation, an economic analysis of this intervention should be undertaken.

Thus, given the need to optimize the VL control program to take advantage of this new intervention, the objective of the present study was to evaluate the cost-effectiveness of insecticide-impregnated collars in dogs in combination with traditional control strategies (the sacrifice of infected dogs and residual chemical control of the vector) in reducing the incidence of canine VL in Brazil.

## METHODS

### General study design 

This was an economic study that included a cost-effectiveness analysis (CEA) conducted from the perspective of the Unified Health System (Sistema Único de Saúde - SUS), using data from the VL Control Program implemented in the municipality of Montes Claros, Minas Gerais, along with results from the project coordinated by Dr. Guilherme Werneck^11^. 

The study period was defined as 2 years (four 6-month cycles, the direct costs of the following three control strategies were evaluated: 1) Canine infection screening + sacrifice, 2) residual chemical control of the vector, and 3) use of insecticide-impregnated dog collars (Scalibor^®^). Taking advantage of prompt access to data from the Municipal Health Department (MHD) of Montes Claros, two areas in this municipality were studied, namely, the intervention and control areas. In the intervention area, all three strategies were evaluated, while in the control area, only strategies 1 and 2 were evaluated. Over the study period, not all spraying cycles were carried out in Montes Claros due to logistical and personnel limitations. Nevertheless, for the purpose of estimating the costs of the residual chemical control of the vector, it was assumed that all spraying cycles were completed. Since this simplification affected both groups equally, the main comparison of interest was not impacted. The characteristics and numerical data of the study areas are presented in Table 1.

**TABLE 1:** Characteristics of the intervention and control areas of Montes Claros, Minas Gerais, studied over 2 years.

Characteristics	**Control area**	**Intervention area**
Population	34,736	32,334
Total number of dogs examined by DPP^®^ and collared	17,716	22,016
Total number of dogs examined by ELISA	2,795	2,255
Total number of sacrificed dogs	980	962
Total number of properties scheduled for spraying	7,695	10,129
Total number of collared dogs	-	22,016

DPP^®^ - Dual-Path Platform.

### Cost estimate and data source

Cost was estimated by microcosting through the identification of human and material resources, unit cost, and number of items or hours of work employed in the implementation of each of the three control strategies. To estimate the cost of human resources, the gross wage proportional to the implementation of the activity, according to the time spent, type of employment, and workload, was considered.

For canine infection screening, the MH recommendations were followed. These state that for initial serological testing, the rapid Dual-Path Platform (DPP^®^) test should be performed at home, followed by the confirmation of positive cases using ELISA (KIT ELISA LVC, Biomanguinhos). Animals positive in both tests were sacrificed. The sacrifice of seropositive dogs in Montes Claros was performed by intravenous injection of a lethal dose of potassium chloride under anesthesia.

The cost estimates of transportation, which included transporting workers, equipment, samples, and animals, were based on the number of liters of gasoline used by the vehicles, according to records from the study period. Three cars and two motorcycles in each study area were exclusively dedicated to the activity canine infection screening and sacrifice daily. For the residual chemical control of the vector activity, two cars were employed, which were also exclusively dedicated to the activity. The insecticide spraying program was intended to be carried out every six months for all areas with a high-moderate VL transmission rate. For this action, the cost of fuel for the vehicles, personal protective equipment, and the insecticide as well as the salaries of the health agents and their supervisors were taken into account. For the use of insecticide-impregnated dog collars, transport costs were not included because collaring was performed at the time of serological testing at home, the cost of which was already accounted for. The number of liters of gasoline consumed by each vehicle was provided by the MHD. The unit costs of cars and motorcycles were obtained from the Institute of Economic Research Foundation. The cost of the depreciation of the vehicles was considered to be 10% per year, for a useful life of 10 years.

To estimate the cost of personal protective equipment, uniforms, diagnostic supplies, insecticides, and field materials, the total amount used in each of the four study cycles and their unit costs were determined, and these numbers were multiplied by each other in each cycle. The cost of insecticides was estimated by multiplying the number of properties sprayed by 100 mL of product, the estimated average volume required for application per property, and the losses that occurred during packaging, filling of the application pump circuit, and transport. It is important to emphasize that the average density of insects can vary depending on several factors, such as the proportion of households and commercial properties present in a given area.

With the exception of the costs of DPP^®^ and ELISA, which were obtained from the MH, all unit values were provided by the MHD.

The number of properties scheduled for residual chemical control of the vector was defined according to surveillance data for Montes Claros following the MH recommendations, which defines the sectors with moderate-high VL transmission levels as priority for the procedure. According to the average number of cases in the last 3 years, the regions with VL transmission were stratified into 1) sporadic, < 2.4, 2) moderate, ≥ 2.4 to < 4.4, and 3) intense, ≥ 4.4 cases. Thus, within the semiannual schedule of activities of the global control program, we calculated one spraying of all properties of each sector with moderate-high levels of LV transmission in each cycle.

For laboratory equipment, according to the recommendation of the Federal Revenue Department’s Depreciation Table, a depreciation rate of 20% per year and a useful life of 5 years were assumed. We also assumed that the municipalities had building infrastructure and professional training; however, expenses on these items were not considered. Table 2 details the items included in the cost estimates of the three control measures, and Supplementary Material details the unit costs of these items.

**TABLE 2:** Types of workers and items included for the three evaluated visceral leishmaniasis control measures.

Control measure	Items included
Canine infection screening + sacrifice	Proportional workers’ wages (Veterinarian, Endemic Diseases Control Agent, Driver, Biologist, and Clinical Pathology Technician), cars, motorcycles, gasoline, clipboards, pencils, pens, erasers, printing, DPP kits, bags, pants, booties, caps, insulated bags, coolers, reusable cool packs, gloves, cords, blood collection tubes, alcohol, needle syringes, cotton, ELISA kits, tips, tubes, pipettes, ELISA readers, microplate washer, incubator, refrigerator, freezer, scale, garbage bag, acepromazine, thiopental, and potassium chloride
Residual chemical control of the vector	Proportional workers’ wages (Endemic Diseases Control Agent and Drivers), cars, gasoline, long-sleeve shirts, pants, boots, full-face masks, filters, nitrile gloves, backpack sprayer, and insecticide
Insecticide-impregnated dog collars	Proportional workers’ wages (Veterinarian and Endemic Diseases Control Agent) and collars

Costs listed in different years were adjusted according to the official inflation rate of the Central Bank of Brazil. All data were entered into spreadsheets created in Microsoft Excel^®^.

### Cost-effectiveness analysis

**FIGURE 1:**
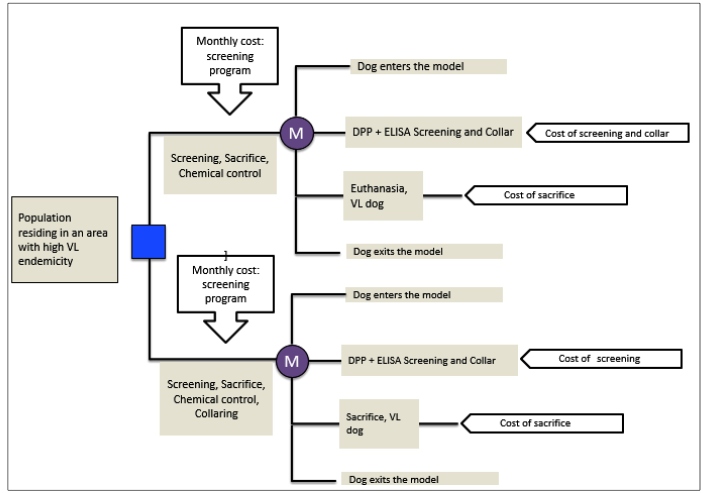
Structure of the Markov model for the decision and cost-effectiveness analysis of the incorporation of the dog-collaring intervention compared to the other VL control measures alone.

To estimate the incremental cost-effectiveness ratio (ICER) in terms of avoided dog sacrifices achieved by the adoption of a large-scale dog screening program with collaring and residual chemical control of the location, an open-cohort Markov decision analysis model was built (Figure 1). Table 1 shows the specifications of this economic analysis.

For the purposes of calculation, two hypothetical target populations, each with 100,000 dog screenings performed, were considered to eliminate differences in the frequencies of events and costs that could be attributed to the different sizes of the target populations. The results of these analyses are expressed as US$/avoided dog sacrifices. The model was built with TreeAge Pro software version 2015 and consisted of four states: 1) Model input (generator of simulated individual dogs), 2) DPP^®^ + ELISA screening and collar (or without collar for the control group), 3) Sacrifice Dog LV, and (4) Model output.

Sensitivity analyses were initially conducted considering alternative effectiveness parameters, such as the risk of canine VL before the implementation of dog collaring. The estimate was not available for the first 6 months (assumed odds ratio [OR] = 1 in the first 6 months, as the base case) but showed decreasing values every 6 months in the original study. This variation in the effectiveness of collaring in the prevention of canine infection was included in the base case of the model, using the following OR values: OR = 1 for month 0-6, OR = 0.54 for months 7-12, OR = 0.48 for months 13-18, and OR= 0.45 for months 19-24^11^.

A probabilistic sensitivity analysis for the cost of collars and effectiveness of collars in the prevention of canine VL, represented by the OR of the occurrence of a case of canine VL, was also conducted. A second-order Monte Carlo analysis was performed, with 10,000 iterations.

For the cost variable, a triangular distribution was assumed, with the value R$ 8.54 as the most likely value and the values R$ 3.82 and R$ 12.82 as the outliers. The lower outlier was derived from the threshold analysis performed in the cost-effectiveness study of collars for the prevention of human VL cases, in which a cost of R$ 3.82 per dog collar was associated with an ICER of R$ 86,626 per avoided human case of VL^11^. The upper outlier (R$ 12.82) for this sensitivity analysis was the approximate unit value of a dog collar, according to the base case of the present study.

Likewise, in the probabilistic sensitivity analysis for the effectiveness variable, the OR of a collared dog acquiring VL was varied over a triangular distribution using 0.54 as the most likely value and 0.45 and 1 as outliers. The first two values were observed in the community intervention, while the third assumed an extreme hypothetical case of lack of collar effectiveness.

Thresholds (threshold analysis) were estimated to establish parameters that would result in more economic measures for the SUS. For interpretation, a willingness-to-pay (WTP) threshold of R$ 86,628 per avoided canine VL case was established (corresponding to 3 times the country’s GDP per capita in 2015). 

## RESULTS

### Direct cost estimate 

The total cost of the program in the area subjected to the interventions currently recommended by the MH (control area) was R$ 1,551,699.80 and in the intervention area, that is, the area where insecticide-impregnated dog collars were implemented in addition to the usual interventions, it was R$ 1,898,190.16 (Table 3). The latter represented an additional expenditure of R$ 346,490.36 over 2 years.

Overall, chemical control was the costliest intervention, representing approximately 73% of the costs in the control area (R$ 1,128,903.20 of the total R$ 1,551,699.80) and 60% of the costs in the intervention area (R$ 1,156,410.36 of the total R$ 1,898,190.16). The intervention with the second-highest cost was canine infection screening with the sacrifice of seropositive dogs, which represented 27% of the expenses of the prevention program in the control area (R$ 424,025.75 of the total R$ 1,551,699.80) and 25% of the expenses in the intervention area (R$ 467,419.79 of the total R$ 1,898,190.16). The implementation of insecticide-impregnated collars represented 15% of the program costs in the intervention area (R$ 282,245.12 of the total R$ 1,843,472.67) (Table 3).

**TABLE 3:** Semiannual costs per cycle, stratified by area (control and intervention), measure, and cost components, incurred by the Visceral Leishmaniasis Control Program in Montes Claros, Minas Gerais from 2012 to 2014.

Activities and cost components	**Control area**					**Intervention area**				
	1^st^	2^nd^	3^rd^	4^th^	Total R$	1^st^	2^nd^	3^rd^	4^th^	Total
	cycle R$	cycle R$	cycle R$	cycle R$	cycle R$	cycle R$	cycle R$	cycle R$	cycle R$	R$
**Canine infection screening - diagnosis by DPP** ^b^ **and ELISA** ^c^										
Human resources	49,797.38	55,379.26	50,008.07	52,742.17	207,926.88	54,318.09	50,707.76	50,305.11	63,496.02	218,826.98
PPE^a^ and uniforms	4,197.06	4,321.52	4,298.40	4,264.46	17,081.44	4,457.02	4,602.72	4,659.10	4,651.24	18,370.08
Transportation	2,468.17	2,262.38	2,581.71	3,169.52	10,481.78	2,432.55	2,296.42	2,846.11	3,654.45	11,229.53
Office supplies	896.11	984.44	968.62	946.05	3,795.22	1,077.07	1,181.58	1,221.67	1,228.46	4,708.78
Materials used in the laboratory	5,711.96	4,871.40	4,499.00	3,504.16	18,586.52	7,058.64	4,729.92	4,546.16	2,985.40	19,320.12
Materials used in the field	29,375.32	32,143.32	31,598.12	30,758.32	123,875.08	35,377.52	38,519.12	39,797.52	39,504.92	153,199.08
**Subtotal**	**92,446.00**	**99,962.32**	**93,953.92**	**95,384.68**	**381,746.92**	**104,720.89**	**102,037.52**	**103,375.67**	**115,520.49**	**425,654.57**
**Sacrifice of seropositive dogs**										
Human resources	8,191.68	6,588.96	4,502.88	5,647.68	24,931.2	10,481.28	7,123.20	3,459.84	3,408.96	24,473.28
PPE^a^ and uniforms	1,041.11	969.29	875.81	927.11	3,813.32	1,143.71	993.23	829.07	864.39	3,830.4
Transportation	1,735.97	1,388.93	1,731.36	1,689.03	6,545.29	1,610.69	1,779.17	1,586.93	1,609.25	6,586.04
Office supplies	27.18	31.38	28.44	32.22	119.22	25.92	25.92	25.50	54.54	131.88
Material used in kennels	2,257.22	1,815.59	1,240.77	1,556.22	6,869.8	2,888.12	1,962.80	953.36	939.34	6,743.62
**Subtotal**	**13,253.16**	**10,794.15**	**8,379.26**	**9,852.26**	**42,278.83**	**16,149.72**	**11,884.32**	**6,854.70**	**6,876.48**	**41,765.22**
**Residual chemical control**										
Human resources	222,315.23	222,315.23	222,315.23	222,315.23	889,260.92	216,135.56	216,135.56	216,135.56	216,135.56	864,542.24
PPE^a^ and uniforms	5,045.57	5,045.57	5,045.57	5,045.57	20,182.28	3,833.72	3,833.72	3,833.72	3,833.72	15,334.88
Transportation	4,589.66	4,589.66	4,589.66	4,589.66	18,358.64	3,393.88	3,393.88	3,393.88	3,393.88	13,575.52
Office supplies	796.51	796.51	796.51	796.51	3,186.04	609.93	609.93	609.93	609.93	2439.72
Insecticide	49,478.85	49,478.85	49,478.85	49,478.85	197,915.4	65,129.5	65,129.5	65,129.5	65,129.5	260.518.0
**Subtotal**	**282,225.8**	**282,225.8**	**282,225.8**	**282,225.8**	**1,128,903.2**	**289,102.59**	**289,102.59**	**289,102.59**	**289,102.59**	**1,156,410.36**
**Dog collaring**										
Human resources	**-**	**-**	**-**	**-**		4,281.45	4,707.3	4,869.65	4,855.20	18,713.60
Collars	**-**	**-**	**-**	**-**		60,292.89	66,289.86	68,576.13	68,372.64	263,531.52
**Subtotal**						**64,574.34**	**70,997.16**	**73,445.78**	**73,227.84**	**282,245.12**
**Total**	**387,924.95**	**387,924.95**	**387,924.95**	**387,924.95**	**1,551,699.8**	**474,547.54**	**474,547.54**	**474,547.54**	**474,547.54**	**1,898,190.16**

^a^personal protective equipment; ^b^Dual-Path Platform (DPP®); ^c^enzyme-linked immunoassay.

The average unit cost of the program per dog considering DPP^®^ and ELISA testing was R$ 20.32, and that of canine infection screening + sacrifice was R$ 459.00.

### Cost-effectiveness analysis

The ICER discounted per avoided dog sacrifice due to the implementation of dog collars was R$ 578.57 (Table 4).

**TABLE 4:** Incremental cost-effectiveness of the collaring program for the prevention of confirmed cases of canine VL indicated for sacrifice: analysis with population sizes of 100,000 inhabitants. Costs and incidence of disease as measured in the municipality of Montes Claros, Minas Gerais.

Strategies	**Cost (R$)**	**Incremental**	**Effectiveness**	**Incremental**	ICER* - no	ICER* - with an
		cost (R$)	(dogs without VL)	effectiveness	discount (R$/case of	overall discount
					avoided canine VL)	of 5%/year
**Canine infection**	3,025,828.39	-	90,052.19	-	-	-
screening +						
euthanasia +						
chemical control						
Canine infection	4,102,934.72	1,077,106.33	91,913.86	1,861.67	569.47	578.57
screening +						
euthanasia +						
chemical control						
+ dog collaring						

* incremental cost-effectiveness ratio.

The univariate sensitivity analyses for the risk measurements of canine VL in locations with the canine collar program (relative risk [RR] of 1 to 0.54 in the first 6 months) and for the cost of the collar (R$ 0 to 13 per dog in the program’s target population) obtained ICER values that were all lower than the WTP threshold defined in the present study (R$ 86,628 per case of avoided dog sacrifice). These analyses assumed that all of the cost components of the programs (dog screening, sacrifice, and residual chemical control) had higher costs in the group that included collaring in the strategy.

**FIGURE 2:**
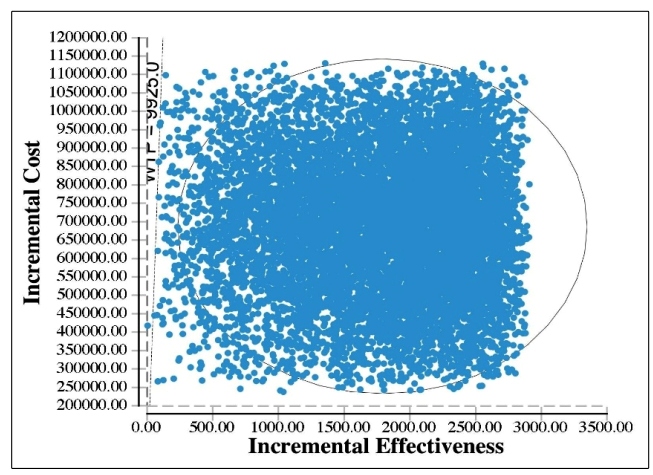
Scatter plot of the probabilistic sensitivity analysis for the parameters collar cost and odds ratio for the prevention of canine cases of visceral leishmaniasis (10,000 iterations). A total of 99.9% of the simulations were below the defined threshold value of 1/3 of the Brazilian GDP per capita (R$ 9,925).

The probabilistic sensitivity analysis of the model focusing on canine outcomes showed that the use of insecticide-impregnated dog collars had a very high probability of being considered highly cost-effective for the outcome of reducing canine VL cases, with almost all of the simulations resulting in ICER values lower than the Brazilian GDP per capita (<R$ 28,875) and 99.9% of the simulations resulting in ICER values lower than 1/3 of the Brazilian GDP per capita (R$ 9,925) (Figure 2).

## DISCUSSION

The estimates and models generated here provide a new perspective on the control measures available for VL, since studies have rarely evaluated the interventions from an economic point of view. However, two published studies, conducted by Camargo-Neves et al. (2011)^12^ and Shimozako et al. (2017)^13^, deserve attention.

In 2010, Camargo-Neves et al. (2011)^12^ evaluated the cost-effectiveness of canine infection screening with sacrifice combined with the use of an insecticide-impregnated collar (Scalibor^®^) compared to canine infection screening with sacrifice + chemical control in a cohort study conducted in Andradina, São Paulo, between 2002 and 2005. The cost of one control cycle using the measures under evaluation (sacrifice + collars) was estimated at US $5,000, and the cost of one control cycle using the comparison measures (sacrifice + chemical control) was estimated at US $14,286. The study showed that canine infection screening combined with the use of insecticide-impregnated collars was a more cost-effective strategy to reduce the canine and human prevalence of VL than sacrifice + chemical insecticide.

Shimozako et al. (2017)^13^, through mathematical modeling considering the temporal dynamics of VL, evaluated the following control activities: dog elimination, insecticide-impregnated collars, dog vaccine, dog treatment, and vector control. Based on the costs already calculated by Camargo-Neves et al. (2004)^14^, the interventions under comparison were estimated as follows: (a) cost to eliminate a dog diagnosed as positive, US $170.71 (collection of samples for testing, performance of indirect immunofluorescence, and sacrifice); (b) cost of the collar, US $12.00 (item made available at the health center and implemented under the responsibility of each owner); (c) cost of dog treatment, US $265.76 (exams + meglumine antimoniate + allopurinol); (d) cost of dog vaccine, US $33.00; and (d) total cost of vector control per household and per mosquito, US $23.14 and US $2.14, respectively. As a result, it was observed that chemical control was the strategy that led to the fastest decrease in the daily number of reported human cases and the greatest reduction in hospital costs, despite requiring the largest investment.

Without the direct data produced by Alves et al. (2020)^11^, the present study could not have been carried out. Through a systematic and exhaustive approach, these authors provided data on the effectiveness of the control measures under evaluation. Although this study was conducted in seven municipalities, direct cost estimates for this CEA were performed considering only Montes Claros, due to logistics and the availability of records. The effectiveness estimates were based on Montes Claros and other municipalities. Thus, although the CEA performed used the best evidence available for Brazil, the data were from a small portion of municipalities affected by VL and should be interpreted with caution. To date, only the rate of *Leishmania*-infected dogs has been evaluated, which is an indirect outcome of the VL Control Program, whose main objective is the reduction of human cases, which is a less frequent outcome and therefore more difficult to measure in the short term.

Regarding the direct individual cost of the control measures, chemical control was the costliest intervention, requiring 73% of the resources in the control area and 60% in the intervention area. Canine infection screening with the subsequent sacrifice of serologically positive dogs was the next costliest measure, requiring 27% and 25% of the resources in the control and intervention areas, respectively. Insecticide-impregnated collars were the least costly measure, consuming 15% of the resources in the intervention area. These results should be interpreted critically because the human population and the number of dogs examined in the control (34,736 and 17,716, respectively) and intervention areas (32,334 and 22,016) were numerically similar but not identical. In general, these results corroborate those observed by Camargo-Neves et al. (2004)^14^, who reported a higher cost for chemical control than for canine infection screening with subsequent sacrifice or environmental management.

In this study, the average cost of serological screening by DPP^®^ and ELISA was estimated at R$ 20.32, and the cost of infection screening + sacrifice was estimated at R$ 459.00. This result is close to that reported by Shimozako et al. (2017)^13^, who estimated the cost of performing canine infection screening + sacrifice at US $170.71. As our two studies had the same base year (2015), cost differences may have been related to the serological tests used, the epidemiological scenario, the sample size, or the data source. It is important to note that the tests included in the cost estimate of canine infection screening in the present study followed the current MH recommendations available in Brazil (2006)^4^.

We performed and interpreted sensitivity analyses considering WTP/disability-adjusted life year (DALY) values proposed by the WHO Commission on Macroeconomics and Health in 2002 (interventions that cost up to the GDP per capita/DALY saved are very cost-effective and less than 3 times the GDP per capita/DALY saved are cost-effective). Undoubtedly, the results of these analyses should be interpreted with caution, as there is no consensus on the threshold under which a health intervention should be considered cost-effective and implemented and, therefore, must be thoroughly discussed. However, it is noteworthy that the economic analyses conducted can be interpreted in the context of a WTP threshold, regardless of the outcome evaluated, even though this threshold is not defined in Brazil.

The addition of insecticide-impregnated collars resulted in an additional expenditure of R$ 404,766.16 over 2 years. In this evaluation, transportation costs were not included in the insecticide-impregnated collar strategy since it was assumed that the dog was collared at the time of serological testing and transportation had therefore been accounted for in the canine infection screening. Thus, the estimated value for this control measure may be underestimated if the application of this strategy alone is considered.

Unlike Camargo-Neves et al. (2011)^12^ and Shimozako et al. (2017)^13^, who individually evaluated the cost-effectiveness of control measures, the purpose of the present study was to perform an economic analysis of two sets of VL control interventions: 1) canine infection screening with the sacrifice of seropositive dogs + chemical control + insecticide-impregnated collars and 2) canine infection screening with the sacrifice of positive dogs + chemical control. Although the results reported here are unprecedented, it should not prevent future analyses from considering the strategies individually.

In the present study, the cost of one insecticide-impregnated collar was estimated at R$ 11.97 per dog, and it was replaced every six months. However, depending on the size of the dog, the same collar can be cut and used by more than one dog, on average two or even three dogs. In this sense, the CEA data concerning the outcome of prevented canine sacrifices and the budgetary impact analysis should be interpreted with caution because they may be underestimated.

There is no predefined method for conducting cost studies of control programs, and therefore, some subjectivity is present when performing this type of analysis. A strength of the present study is the reliability of the estimated costs, provided by the Montes Claros MHD and based on detailed records of the consumption and cost of each item used in the program, both for the control and intervention areas. Thus, all estimates were performed considering the available data to more accurately translate the true costs over the 2 years of evaluation.

The CEAs conducted indicated that the collaring program seems to be highly cost-effective in preventing canine VL (ICER of approximately R$ 578 per avoided dog sacrifice). Regarding the probabilistic sensitivity analysis for the outcome of canine VL, it must be noted that even though the values for the prevention of canine cases can be considered highly cost-effective, there is no direct translation to human outcomes. Since dogs are inserted in the human VL transmission chain but are not directly linked, it is expected that the ICER of the impregnated dog collar program for the reduction of human VL cases will be substantially higher (therefore less cost-effective) and accompanied by a greater degree of uncertainty.

This study intended to reliably evaluate the direct cost of the VL control program implemented in Montes Claros over the 2-year interventional study period. The different control strategies were compared assuming that all spraying cycles were carried out as planned. In actuality, however, not all cycles were performed completely. Despite being a limitation, this simplification affected both the control and intervention groups equally, and therefore should not have impacted the comparison between interventions. Another important limitation is the underestimation of the effectiveness of the insecticide spraying program since it is often not performed as planned due to shortages of supplies, reallocation of health workers, and policy interruptions. Since this is a reality common to most endemic regions in developing countries, this would likely affect other studies addressing such interventions. 

Previous studies have addresseding the effectiveness of insecticide-impregnated collars to control canine VL. In a study conducted in the municipality of Governador Valadares, Minas Gerais, Brazil, Coura-Vital et al. (2018)^15^ concluded that the uninterrupted use of deltamethrin-impregnated collars reduced canine infection by *Leishmania*. Recently, a systematic review with meta-analysis conducted by Yimam and Mohebali (2020)^16^ also confirmed that the use of insecticide-impregnated dog collars can reduce the risk of VL caused by*L. infantum*. 

The results of the present study provide support for the decision of the Brazilian MH in 2019 to provide insecticide-impregnated collars for the control of canine VL in a pilot project. It is important to highlight that the results presented here must be analyzed with caution, taking into account that the conclusions are based on data from a single Brazilian municipality. The collars are distributed free by the government, from household to household, accompanied by a routine blood test that is performed regularly to diagnose the evolution of the disease in the canine community. A project to evaluate the implementation of large-scale collar use will also be conducted across the country.

Despite this study’s contributions, it is important to note that the modeling used is a simplified representation of reality. Due to this simplification, important and current factors related to the dynamics of the disease may not have been taken into account. For example, the impact of seasonal and climatic changes on the vector population, asymptomatic infection in humans and dogs, and the commercial availability of treatments and vaccines for dogs.
