# Supplementary material for: Cost-effectiveness of a canine visceral leishmaniasis control program in Brazil based on insecticide-impregnated collars
Source: Rev Soc Bras Med Trop. 2020 Dec 11;53:e20200680. doi: 10.1590/0037-8682-0680-2020 (PMC7747826; doi:10.1590/0037-8682-0680-2020)
Supplement: Supplementary file 1 [file 1678-9849-rsbmt-53-e20200680-suppl1.pdf]

SUPPLEMENTARY MATERIAL. Unit cost of each item included in the direct cost estimates performed for visceral leishmaniasis, base year, 2015..

Item	Cost (R$)
Wage/Veterinarian, 30 hours per week	2501.58
Wage/Biologist, 20 hours per week	1667.73
Wage/Clinical Pathology Technician, 30 hours per week	1468.35
Wage/Endemic Diseases Control Agent and Driver, 40 hours per week	1171.17
Clipboard, Stalo brand	2.03
Pencil, CIS HB brand	0.11
Pen, Bic Cristal brand	1.08
Eraser, Zapp brand	0.27
Printing	0.07
Dual-Path Platform (DPP®), Biomanguinhos	6.80
ELISA Reaction, KIT ELISA LVC, Biomanguinhos	5.24
Bag, locally made/no brand	36.40
Long sleeve shirt, locally made/no brand	12.49
Pants, locally made/no brand	45.35
Boots, Kadesh brand	48.48
Cap, locally made/no brand	6.17
Insulated bag, Western Soprano brand	35.20
Cooler, Thermos brand	97.20
Reusable cool pack, Gelo Tech brand	3.84
Gloves (pair), Supermax brand	0.30
Nitrile gloves (pair), Nitrilinea brand	5.50
Mask, Descarpack brand	0.08
Apron, locally made/no brand	12.50
Cord with ring, Nugard brand	3.80
5 ml blood collection tube, Coral brand	0.24
Alcohol (liter), Itajá brand	3.25
Needle syringe, SR brand	0.28
Cotton (500 g), Nathálya brand	7.08
Tip, Kasvi brand	0.01
Eppendorf tube, Kasvi brand	0.03
Garbage bag, brand not reported	0.28
Blood collection set, Solidor brand	0.15
Acepromazine (20 ml ampoule), Syntec brand	31.90
Thiopental (1 g bottle), Cristália brand	36.90
Potassium chloride, brand not reported	45.00
3 M full face mask filter, Air Safety brand	40.00
Alpha-cypermethrin (1 liter), brand not reported	64.36
Insecticide-impregnated collar, brand Scalibor®	11.97
